# Long Dynamic β1–β2 Loops in *M. tb* MazF Toxins Affect the Interaction Modes and Strengths of the Toxin–Antitoxin Pairs

**DOI:** 10.3390/ijms25179630

**Published:** 2024-09-05

**Authors:** Ziyun Tang, Pengcheng Jiang, Wei Xie

**Affiliations:** 1 MOE Key Laboratory of Gene Function and Regulation, State Key Laboratory for Biocontrol, School of Life Sciences, Sun Yat-Sen University, Guangzhou 510275, China; tangzy9@mail2.sysu.edu.cn (Z.T.); jiangpch3@mail2.sysu.edu.cn (P.J.); 2 Innovation Center for Evolutionary Synthetic Biology, School of Life Sciences, Sun Yat-Sen University, Guangzhou 510275, China

**Keywords:** tuberculosis, *Mycobacterium tuberculosis*, MazEF, TA systems, crystal structures, MazF-mt3, conformational changes

## Abstract

Tuberculosis is a worldwide plague caused by the pathogen *Mycobacterium tuberculosis* (*M. tb*). Toxin–antitoxin (TA) systems are genetic elements abundantly present in prokaryotic organisms and regulate important cellular processes. MazEF is a TA system implicated in the formation of “persisters cells” of *M. tb*, which contain more than 10 such members. However, the exact function and inhibition mode of each MazF are not fully understood. Here we report crystal structures of MazF-mt3 in its apo form and in complex with the C-terminal half of MazE-mt3. Structural analysis suggested that two long but disordered β1–β2 loops would interfere with the binding of the cognate MazE-mt3 antitoxin. Similar loops are also present in the MazF-mt1 and -mt9 but are sustainably shortened in other *M. tb* MazF members, and these TA pairs behave distinctly in terms of their binding modes and their RNase activities. Systematic crystallographic and biochemical studies further revealed that the biochemical activities of *M. tb* toxins were combined results between the interferences from the characteristic loops and the electrostatic interactions between the cognate TA pairs. This study provides structural insight into the binding mode and the inhibition mechanism of the MazE/F TA pairs, which facilitate the structure-based peptide designs.

## 1. Introduction

Tuberculosis (TB) is the second leading cause of death from infectious diseases worldwide, following COVID-19 since 2022 [1]. Discovered by Robert Koch in 1882, the pathogen *Mycobacterium tuberculosis* (*M. tb*) is responsible for the pathogenicity of TB [2,3]. Global statistics of the past decade from the WHO reveal that the rise of drug-resistant *M. tb* strains has notably decreased the cure rates of TB [1]. At present, the only approved vaccine for TB prevention is Bacille Calmette–Guérin (BCG), which is derived from bovine rather than human sources [4,5].

Toxin–antitoxin (TA) systems were first identified on plasmids in the 1980s [6] and have since been found to extensively exist on bacterial chromosomes [7,8,9]. Typically consisting of two or three genes within a single operon, TA systems include a stable toxin gene encoding a protein and an antitoxin gene encoding an unstable noncoding RNA or protein [10,11,12,13]. TA systems are crucial in bacterial physiology, especially in pathogens such as *M. tb*, affecting their growth, stress responses, and adaptation to host environments [14]. Currently, eight types of TA systems have been identified, categorized based on the nature of the antitoxin and the mechanism of interaction between the toxin and antitoxin [10,13,15,16]. Type II TA systems are the most common TA systems and feature both the toxin and antitoxin as proteins. Classic examples of type II TA systems include YefM-YoeB [17], VapBC (Virulence associated protein BC) [18], MazEF [19], and RelBE [20].

In *M. tb*, approximately 90 TA systems have been identified, with most belonging to the VapBC and MazEF families [21]. In contrast, the closely related non-pathogenic bacterium *Mycobacterium smegmatis* possesses only five TA systems, highlighting the potential role of TA systems in pathogenicity [22]. While these systems are widespread in bacteria, they are absent from the human genome, making them promising targets for drug development [23]. Zhu et al. have identified homologs of *Escherichia coli mazf* in the *M. tb* (H37Rv) genome through BLAST searches, discovering putative *mazef* members termed *mazef-mt1-7* [24]. To date, more than 10 members of the MazEF family have been identified in *M. tb* [11].

Studies of antibiotic-induced *M. tb* transcriptomes show upregulation of at least 10 TA systems, suggesting their potential role in bacterial persistence [25]. Overexpression of MazF-mt1, MazF-mt3, or MazF-mt6 in *Mycobacterium bovis* BCG induces growth inhibition. Strains lacking multiple *mazf* genes exhibit reduced survival under oxidative stress, nutritional limitations, and inside macrophages [14], but the direct roles of these toxins in the infection processes remains unclear [11]. Differential expression of MazEF-mt3 and MazEF-mt6 correlates with drug-sensitive and drug-resistant strains, underscoring the role of specific TA systems in bacterial adaptation and survival [26]. The transition from rapid growth to non-replicating persistence (NRP) is a hallmark of chronic tuberculosis infection, involving complex biological changes [27]. Regulatory proteins such as Soj_Mtb_ (encoded by *rv1708*) influence cell cycle regulation during septation, potentially coordinating stress responses and NRP transitions [28].

All the MazF toxins from the family are RNases, capable of cleaving various types of RNA including mRNA, tRNA, or rRNA. In-vivo extension experiments with heterologous expressions of EcMazF [29] and MazF-mt1 [24] indicated their cleavage specificity as mRNA interferases, both recognizing trinucleotide bases (the A↓CA and U↓AC motifs, respectively). However, the cleavage activity of MazF-mt3 was limited, possibly due to its recognition motif being longer than three bases. In-vitro extension experiments using bacteriophage MS2 RNA as a substrate, assisted by the *E. coli* CspA protein for secondary-structure elimination, revealed that MazF-mt3 recognizes the consensus motifs UU↓CCU or CU↓CCU [30]. To better reflect the specificity of MazF-mt3 in *M. tb*, the so-called MORE RNA-seq was developed, which identified the cleavage sequence as U↓CCUU. Furthermore, it also discovered two cleavage sites on the 23S and 16S rRNAs. Subsequent extension experiments confirmed that MazF-mt3 targets helix/loop 70 of 23S rRNA. Specifically, it cleaves at ^1537^U↓CCUU^1541^ of the anti-Shine–Dalgarno (aSD) sequence of 16S rRNA of the 70S ribosome [31]. 

The MazF toxin structures from various bacteria, such as *Bacillus subtilis*, *Escherichia coli*, and *Staphylococcus aureus*, were among the early determined structures in the MazF family (PDBs 4ME7, 5CO7, 5CKB, 5CKD, 5CKF, 5CKH, 3NFC, 2MF2, 4MZT, 4MZM, 4MZP, and 4OF1) [32,33,34,35]. In the past decade, the *M. tb* MazF toxin structures in various forms were also successively solved. Particularly, a series of structures of *M. tb* MazEF TA complexes were determined as well, (PDBs 5XE3, 6L29, 6L2A, 6KYT, 6KYS, 7DU4, and 7DU5) [36,37,38]. Analysis of MazE antitoxin sequences and available structures shows that they consist of two domains: an N-terminal DNA binding domain (DBD) and a C-terminal intrinsically disordered region involved in toxin binding [39,40,41]. Lastly, the structures of toxins in complex with their nucleic acid substrates were also determined (PDBs 5HJZ, 5HK3, 5HKC, and 5HK0). However, the structures of the nucleic acid-bound *M. tb* MazFs were only deposited in the PDB database and released later, but the related studies were not published.

Previously, we determined the crystal structures of *M. tb* MazF-mt9 and -mt1 in their apo- and antitoxin-bound forms (PDBs 5WYG, 6A6X, 6KYS, 6L29, 6KYT) [36,37,42,43] and characterized the association modes between the cognate and noncognate toxin–antitoxin pairs. We discovered that MazF-mt1 possessed two long loops between the β1 and β2 strands (defined as the β1–β2 loop hereinafter) with inter-subunit interactions within the MazF-mt1 dimer, and the binding of the antitoxin requires an “unhook” between the two subunits. The loops in MazF-mt1 cross-interact with each other between the two subunits, and the binding of the antitoxin unlocks these loops. In contrast, the corresponding loops in MazF-mt9 remain disordered, regardless of the binding of MazE-mt9. We also determined the cocrystal structures of the two toxins complexed with their antitoxin peptides (PDBs 7DU4 and 7DU5) [37]. Interestingly, MazF-mt9 binds its C-terminal helix while MazF-mt1 binds its second-to-last helix. Sequence analysis indicated that MazF-mt3 (Rv1991c) also contains a long β1–β2 loop, and we wonder if it would behave similarly. In this study, we solved two crystal structures of MazF-mt3 in two forms, which together revealed the distinct inhibition mechanisms adopted by these MazF members. This study provides structural insight into the binding mode between the MazE/F-mt3 TA pair, and in-depth mechanistic studies revealed the structural basis for different antitoxin–toxin pairings.

## 2. Results

### 2.1. Structures of Apo-MazF-mt3

**Figure 1:**
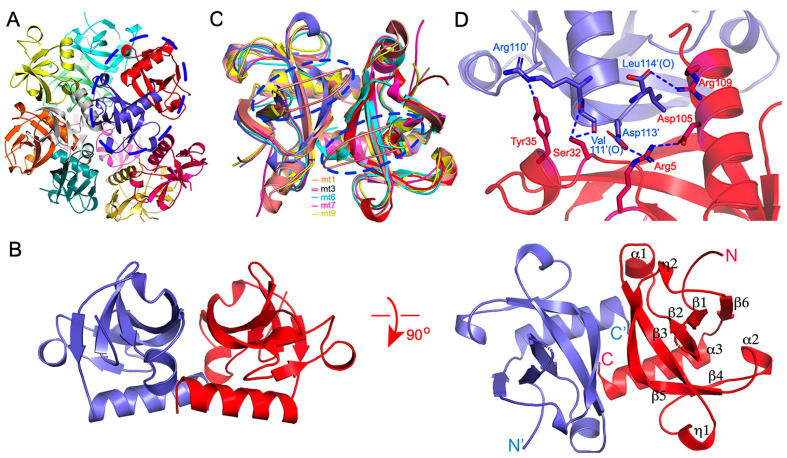
Overall structure of MazF-mt3. (**A**) The MazF-mt3 assembly in the asymmetric unit in the ribbon rendition. The 12 monomers are color coded, forming 6 dimers. The dimer for the close-up view in (**B**) is circled. (**B**) The representative MazF-mt3 dimer in two orthogonal views. The two subunits are colored red and slate, respectively, The N- and C-termini are indicated, and the secondary structure elements of MazF-mt3 are labeled. (**C**) Structural comparison between MazF-mt3 (red and slate) and other toxin members in the *M. tb* MazF family, which include MazF-mt1 (PDB 6KYS, orange), mt6 (PDB 5CCA, cyan), mt7 (PDB 5XE2, pink), and mt9 (PDB 5WYG, yellow). The loop regions are circled by the broken ovals. (**D**) Close-up view of the dimer interface at the C-termini. The side chains of the key interfacial residues are shown as sticks and labeled. The hydrogen-bonding and salt-bridge interactions are indicated by the blue dashed lines.

To purify the recombinant MazF-mt3 protein, we first attempted its heterologous expression in *E. coli* with an N- or C- terminal 6×His tag. However, the purified protein was rather unstable and was refractory to crystallization. We then cloned and expressed the MBP–MazF-mt3 fusion protein in *E. coli*, with a PreScisscion protease (Cytiva) recognition site inserted between MBP and MazF-mt3. After the expression and removal of the fusion partner, the resulting protein consisted of a total of 118 residues, including the GPEL tetrapeptide preceding the initiating methionine residue. We next crystallized and solved the apo structure of MazF-mt3 at 3.3 Å, using the alphafold 2-predicted structure as the search model for the molecular replacement. The space group is *P*4_3_2_1_2, and the unit cell is rather large, with the three dimensions measuring 110.8, 110.8, and 271.2 Å. There are 12 MazF-mt3 monomers present in the asymmetric unit (ASU), assembling into six dimers (Figure 1A). During the refinement, noncrystallographic symmetry (NCS) was applied, resulting in the *R_free_* and *R_work_* of the final model of 0.279 and 0.242, respectively (Table 1). Most chains resolved nearly all the residues except for an internal proline-rich loop of a 9-residue length between β1 and β2.

We next will choose the A/G dimer for structural descriptions due to their more complete structure. The two monomers were closely associated with each other and were also very similar in structure (a 0.4 Å RMSD over 105 Cαs, Figure 1B and Figure S1). Each subunit displays a typical MazF fold, featuring a central antiparallel β-sheet composed of six strands (Figure 1B). The helices are mostly short, except for the last helix α3, which also forms part of the dimer interface. The formation of the dimer buries a surface area of 2853.8 Å^2^.

### 2.2. Sequence and Structure Comparison with of Other Members of the MazEF Family

The structures of several MazF members have been solved in various forms, namely MazF-mt1, mt6, mt7, and mt9 (PDB entries 5HJZ, 6L29, 6L2A, 6KYT, 6KYS, 7DU5, 5UCT, 5CCA, 5XE2, 5XE3, 5WYG, 6A6X, and 7DU4) [36,37,38,42,43,44], enabling a comparative analysis of their structural similarities and differences. The overall shapes of apo-MazFs resemble each other (Figure 1C). However, the cross-subunit loops of MazF-mt1 are quite unique while their counterparts in MazF-mt3 and mt9 are disordered. In comparison, MazF-mt6 and mt7 lack such long loops, which are simply replaced by shorter β-turn motifs. Another notable feature is that α3 in MazF-mt3 is rich in Arg (Arg106, Arg109−110), which forms inter-subunit salt bridges or hydrogen bonds (Figure 1D). Specifically, the guanidino group of Arg109 establishes an unidentate salt bridge with the free carboxylate group of the last residue Leu114′ (the last residue of the chain and apostrophe indicates residues from the other protomer). Asp105 on the same side of the helix forms an intrasubunit salt bridge with Arg5 which in turn forms an intersubunit salt bridge with Asp113′. These residues show well-defined densities, suggesting the robustness of the interactions (Figure S2). On the other hand, Arg110′ and Val111′ of α3′ form an intersubunit hydrogen bond with Tyr35 and Ser32, respectively. These interactions contribute to the stabilization of the dimer interface of MazF-mt3 and may be protein-specific because the residues involved are not conserved in the homologs.

### 2.3. RNA Cleavage Activity and Key Residues Involved

We next explored the catalytic features of MazF-mt3. Zhu et al. reported that the ss-RNA substrate 5′-AGUCUCCUUUC-3′ harboring the putative recognition site would serve as a suitable substrate for this toxin [30]. We utilized a 13-nt FAM-labeled RNA substrate (FAM-AAGUCUCCUUCAG) for the activity experiments and the optimal pH, salt concentration, and temperature of MazF-mt3 were tested. We found that the best reaction buffer was 20 mM Tris-HCl pH 8.0 and 150 mM NaCl. When the reaction was incubated at the molar ratio of 1:6 (enzyme/substrate) at 37 °C, the reaction could be just finished in 30 min (Figure S3).

**Figure 2:**
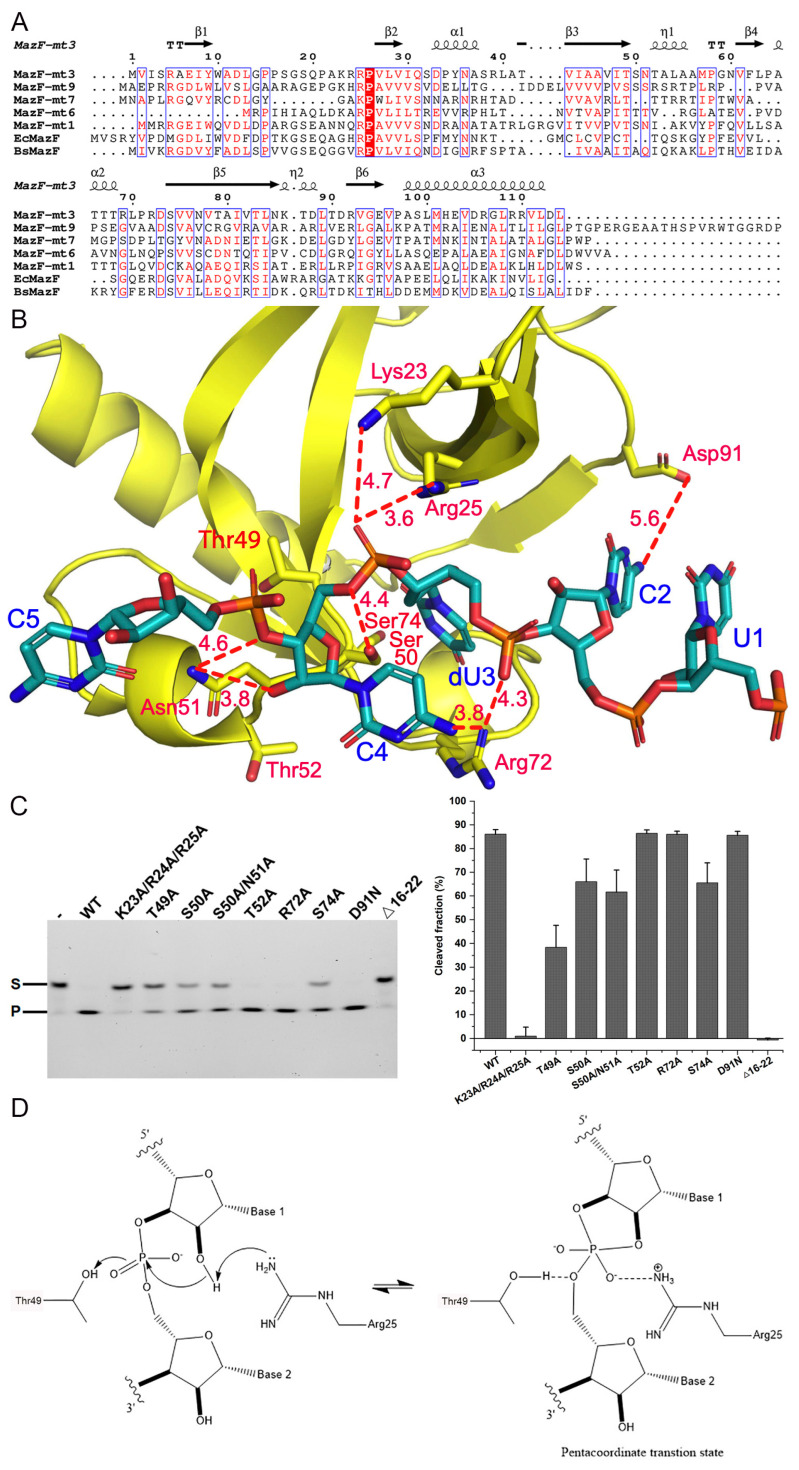
The biochemical features of MazF-mt3 catalysis. (**A**) Multiple sequence alignments of MazF-mt3 and other MazF members, and the secondary structure elements of MazF-mt3 were indicated on the top of the alignment. Identical residues in sequences are on a red background, and similar residues are in red. (**B**) The pre-catalytic model of MazF-mt3 bound by a 5-nt RNA oligonucleotide. The potential key interfacial residues for contacts or catalysis (within a 6-Å distance from the RNA substrate) were indicated and shown as sticks. The distances between the cortical atoms are shown beside the dashed lines. (**C**) The activity tests of MazF-mt3 and variants, analyzed by cleavage of FAM labeled-RNA substrate of 13 nt. S: substrate; P: product. Left: the gel-electrophoresis of the cleavage assays; Right: the quantification of the enzymatic activities, with the vertical axis indicating the cleaved fractions of the substrate. Error bars are standard deviation (s.d.) (n = 3 biological replicates). (**D**) The proposed mechanism for MazF-mt3 catalysis.

Previous studies have identified several key residues for the RNA cleavage activity of MazFs, and a sequence alignment showed that these residues are quite conserved, especially for Thr49 and Arg25 (MazF-mt3 numbering, Figure 2A). The sequence identities and similarities were 34% and 50%, respectively, for MazF-mt1, 23% and 41%, respectively, for mt6, 27% and 43%, respectively, for mt7, and 38% and 58%, respectively, for BsMazF, etc., across the full sequences. The equivalent residues of MazF-mt3/Thr49 in *E. coli* and *B. subtilis* MazFs (Thr52 and Thr48, respectively) are believed to play a role in stabilizing the buildup of negative charge in the bipyramidal transition state while Arg25 fulfills a dual general base/general acid role (corresponding to Arg29 in EcMazF and Arg25 in BsMazF, respectively) [32,33]. Additionally, we created a model by superimposing the protein component from the MazF-mt3 complex (chain A of PDB 5HK0) onto apo-MazF-mt3 (PDB 9IKD) and found that residues Thr49, Ser50, Thr52, Arg72, Ser74, and Asp91 were in close ranges from the 5-nt RNA substrate (Figure 2B). The RNA is composed entirely of pyrimidines, and the key uracil (dU3), whose 3′-phosphodiester bond was set to be cleaved, was replaced by a deoxyuridine (dU). This base was flipped inside into the recognition pocket with its O3P atom only 4.4 Å from Ser50. The O2P atom of dU3 is 3.6 Å and 4.7 Å from Arg25 and Lys23, respectively, while its phosphodiester backbone is recognized by Arg72, which also makes a potential long hydrogen bond with the C4 base (3.8 Å). Additionally, Asn51 makes a salt bridge with the C4 backbone phosphate and the C2 base is within a 6.0-Å distance from Asp71. Hence, we created the T49A, S50A, T52A, R72A, S74A, and D91N single mutations as well as the S50A/N51A and K23A/R24A/R25A multi-mutations for further activity investigations. These mutants were expressed and purified to homogeneity (Figure S4). Before these mutants were subjected to the RNA cleavage assays, thermal shift assays (TSA) were conducted to assess the effects of the mutations on the folding of the variants (Figure S5). The results showed that the mutations near the active site residue Thr49 (i.e., Ser50, Asn51, and Thr52) could perturb the protein structure to some extent (a reduction in the Tm value ~4–5 °C, Figure S5), while the other mutants maintained their basic folds. Correspondingly, the S50A and its double mutant S50A/N51A lost ~1/5 of their activities, as did the S74A mutant (Figure 2C). Additionally, the T49A mutation reduced the enzymatic activity by half, while the triple mutant K23A/R24A/R25A barely had any activity. In comparison, the remaining mutants were as active as the WT enzyme.

Therefore, the transesterification reaction would proceed via a nucleophilic displacement at the phosphorus of the 5′-leaving group by the incoming 2′-hydroxyl, with the formation of a pentavalent transition state [45]. Our activity assays suggest that Arg25 acts as the putative general acid/base while Thr49 possibly interacts with the scissile chain, stabilizing the negative charge in the transition state (Figure 2D).

### 2.4. Structures of the MazF-mt3 in Complex of Its Antitoxin Fragment

**Figure 3:**
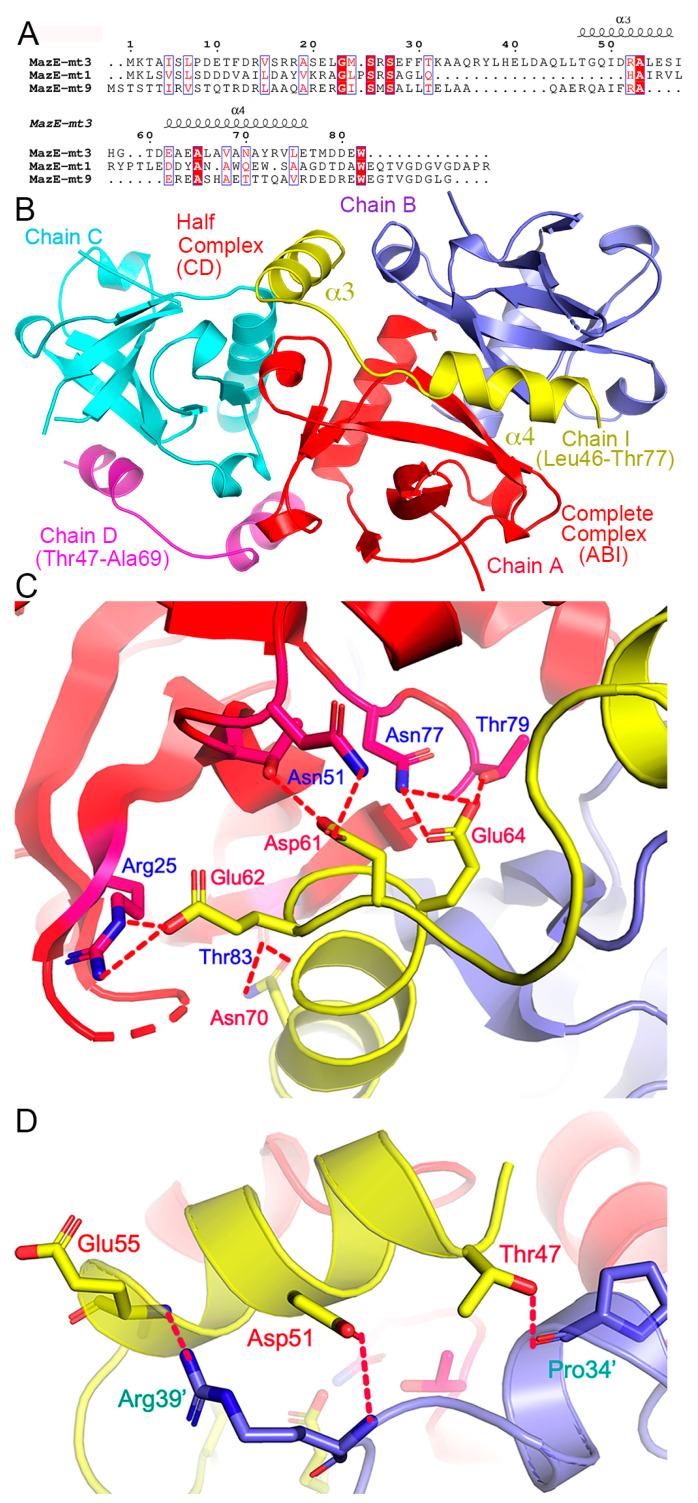
Overall structure of the MazEF-mt3 TA complex. (**A**) The multiple sequence alignment of MazE-mt3, -mt1 and -mt9 and the secondary structure elements of MazE-mt3 were indicated on the top of the alignment (based on the structure of PDB 8ZWS). Identical residues in sequences are on a red background, and similar residues are in red. (**B**) The MazF-mt3 assembly in the asymmetric unit in the ribbon rendition. The complete and half complexes are labeled. The two fragments of MazE-mt3 are colored yellow and magenta, respectively. (**C**,**D**) Close-up view of the TA complex interface at the Asp61-Glu64 fragment (**C**); and at the α3-helix (**D**). The side chains of the key interfacial residues are shown as sticks and labeled. The hydrogen-bonding and salt-bridge interactions are indicated by the red dashed lines (cutoff distance: 3.65 Å).

To reveal the inhibition of the MazF-mt3 activity by its antitoxin, we attempted a cocrystallization of the toxin with the full-length MazF-mt3 proteins, or its truncated versions corresponding to MazE-mt3^46−82^ (the α3-α4 helices), MazE-mt3^60−82^ (α4), and MazE-mt3^47−59^ (α3), etc. (Figure 3A). These proteins or peptides were either recombinantly expressed or chemically synthesized. The two components of the complex were isolated first in their pure forms and then mixed together according to a 2:1 molar ratio (toxin/antitoxin). Through extensive trials, only the MazE-mt3^46−82^ peptide produced suitable crystals for structure determination. The final model contained three toxin monomers (chains A, B, and C) and two antitoxin monomers (chains D and I) with a space group *P*3_1_21. These molecules formed peculiar combinations: while the toxin dimer comprising chains A/B complexed with one of the antitoxins (chain I) in a stoichiometry of 2:1 (named “the complete complex” hereinafter), the remaining toxin (chain C) associated with the other antitoxin (chain D) in a stoichiometry of 1:1 (“the half complex”) (Figure 3B). The β1–β2 loops of the three toxin molecules were still disordered while chains I and D resolved residues Leu46-Thr77 and Thr47-Ala69 of the antitoxins, respectively. Of note, the partial helix of chain D coincides with the two-fold crystallographic symmetry axis, and a symmetry operation would generate the toxin dimer (Figure S6). In other words, the resolved helical part Glu62-Ala69 made crystal contacts with its counterpart from a symmetry mate (broken oval in Figure S6). Since the incomplete complex CD resulted from crystal packing, we will focus our discussion on the complete complex.

The binding of the peptide did not change the structure of the toxin dimer, which is common among the MazEF members (Figure S7). Upon the binding of the antitoxin, the unstructured loops in MazF-mt3 remained partially disordered (except for 2–3 additional resolved residues). The peptide formed two short helices (α3-α4, Figure 3B) situated across the deep crevice formed by the dimer, and the α4-helix was located in the proximity to the loops. Extensive protein–protein interactions including salt bridges, hydrogen bonds, stacking, and hydrophobic interactions were observed. The total buried surface is 2392.8 Å^2^ between the MazF-mt3 dimer and its contacting MazE-mt3 monomer. Due to the moderate resolution of our complex structure, some side chains of the peptide residues connecting the two helices and at the C-terminus were not visible and therefore not modeled. The interactions are listed in Table 2 and are mainly concentrated in the α3-helix and α3-α4 loop in MazE-mt3 (Figure 3B). Of note, charged residues Asp61, Glu62, and Glu64 on the α3-α4 loop each made 2–3 hydrogen bonds or salt bridges with the toxin, all via their side chains, while Asn70 made hydrogen bond contacts with the backbone of Thr83 (Figure 3C). On the other hand, the α3 residues Thr47, Asp51, and Glu55 all made hydrogen bonds with the other monomer of the toxin (Figure 3D). Taken together, most intermolecular contacts are from the α3-helix and the α3-α4 loop.

### 2.5. The Role of the β1–β2 Loop in the Toxin–Antitoxin Interactions

Sequence and structural alignments showed that the MazF-mt1/3/9 toxins possess the β1–β2 long loops while their counterparts in other toxins are relatively short. These long loops display distinct structures as well (Figure 1C). Comparison of the antitoxin binding profiles between MazF-mt1 revealed that the unlocking of the intertwined loops would require an energy penalty in the binding of the α4-helix (MazF-mt3 numbering, corresponding to residues Ala57-Gly72). We investigated how MazF-mt3 behaves in this context.

**Figure 4:**
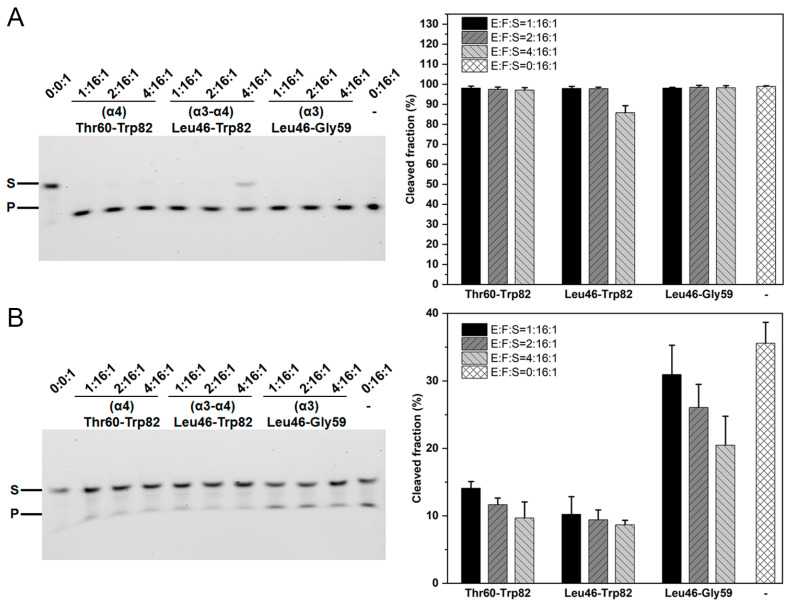
The cleavage activities of MazF-mt3 and mutant in the presence of inhibiting peptides. (**A**,**B**) The inhibitory effects of peptides (corresponding to the α3 (Leu46-Gly59), α4 (Thr60-Trp82) and α3–α4 helices (Leu46-Trp82) of MazE-mt3) on the RNase activities of WT MazF-mt3 (**A**); and the MazF-mt3 (Δ16–22) mutant (**B**). The numbers above the lanes indicated the molar ratios of peptide/toxin/RNA. Left: the gel-electrophoresis of the cleavage assays; Right: the quantification of the enzymatic activities, with the vertical axis indicating the cleaved fractions of the substrate.

The WT MazF-mt3 enzyme was quite active under the testing conditions (in a 15-fold excess), leaving no RNA substrate uncleaved during the reaction period. However, we found that the full-length MazE-mt3 could fully suppress the cleavage activity of MazF-mt3 when the molar ratio was raised to 2:1 (antitoxin/toxin) (Figure S8). In contrast, the last helix (α4, fragment Thr60-Trp82) or the second-to-last helix (α3, fragment Leu46-Gly59) of the antitoxin exhibited minimal inhibitory effects. It was the peptide corresponding to the last two helices (α3-α4, i.e., Leu46-Trp82) with a molar ratio of 1:4 (antitoxin/toxin) that reduced the cleavage of the enzyme by 15% (Figure 4A). We then removed the loop residues Pro16–Ala22 and further tested the consequences resulting from the loop loss. The mutant MazF-mt3(Δ16–22) itself showed reduced activity toward the same RNA substrate and only ~a half of the substrate would be cleaved at the identical condition. Additionally, the presence of the α4-helix at a ratio of 1:16 inhibited more than 60% of the activity of the MUT enzyme, while at a ratio of 1:4, its RNase activity would be further reduced to 1/4, a result similar to the combined α3–α4 helices (Figure 4B). The result is reasonable because the presence of the α4-helix of MazE would bring both pros and cons to the binding to the WT toxin enzyme, while α4 may make some contacts with the toxin to provide a greater affinity, which may be offset by the inference from the loops. In contrast, the truncation mutant of MazF-mt3 (Δ16–22) did not incur this penalty because the dynamic loops were already deleted. This trend would be more evident when we normalize the activity of the truncation mutant to 100% (Figure S9). From the biochemical results, we inferred that the binding of MazE-mt3 to MazF-mt3 is mainly attributed to the last two helices of the antitoxin while the inhibitory effects mainly stem from the hindrances of the loops on the last helix. The long structurally disordered loops of the toxin somehow interfere with the binding of α4 and also require an energy “penalty” similar to what was observed in the MazF-mt1 scenario [36]. However, once the loops are removed, the helix would show an inhibition role as strong as that of the full-length antitoxin, and this scenario has been observed in the MazF-mt9 studies, where the last helix exhibited an affinity equal to that of the intact MazE-mt9 antitoxin [37].

To further test our hypothesis, we conducted similar experiments on the WT MazF-mt1 and its loop-truncated mutants. However, the deletion mutant did not express in *E. coli*. Nevertheless, we also tested this hypothesis on MazF-mt6, which has very short loops. It showed endoribonuclease activity toward the RNA substrate GGUUCCUGC (specific for the UU↓CCU motif) [46]. However, the binding of the α4-helix of MazE-mt6 alone would render the toxin inactive, which is another case in point (Figure S10).

### 2.6. The Effects of TA Charge Complementation on Binding

**Figure 5:**
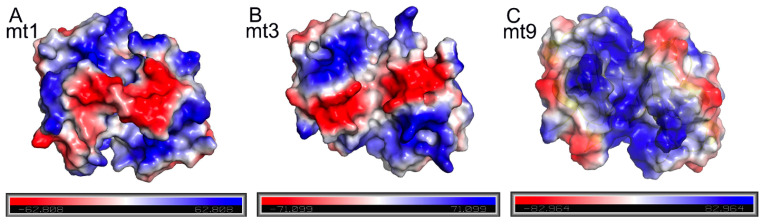
The distinct surface charge distribution patterns of different *M. tb* MazFs with long β1–β2 loops. (**A**–**C**) The charge distribution of the MazF-mt1 dimer (**A**); of the MazF-mt3 dimer (**B**); and the MazF-mt9 dimer (**C**). Note that the loops are either manually removed (mt1) or naturally disordered (mt3 and mt9) to ensure the same regions are examined.

In contrast to the steric hindrance from the loops of toxins that necessitates an energy penalty for the antitoxin to bind, the favorable interactions between the TA pairs play positive roles in their mutual affinities. Besides the above-mentioned hydrogen bonds of the complex, other interactions, especially electrostatic ones, also contribute substantially. Interestingly, we found that the structures of various MazFs show different surface charge distribution patterns, suggesting distinct electrostatic interaction patterns between the toxins and their cognate antitoxins (Figure 5). Notably, the dimer interface of MazF-mt9 is quite positively charged, corresponding to a high theoretical isoelectric point (pI) value of the protein (higher than those of MazF-mt1 and -mt3, Table 3). Here we used the same naming system for the secondary structural elements according to the sequence homologies in Figure 3A. Therefore, it will be highly positively charged at a neutral or slightly basic pH. On the other hand, the pI values of antitoxins are typically low, hence exhibiting negatively charged natures. For instance, the pI of the full-length MazE-mt9 is 5.1, whereas the pI values for the α3, α4, and α3–α4 helices are 6.8, 3.7, and 4.4, respectively. Consequently, α4 binds to the toxin more easily than α3 alone, or α3–α4 combined, because it shows a lower pI value than the latter two and suffers a smaller penalty from the intrinsically disordered loops. For this reason, we successfully cocrystallized the MazE-mt9/α4-MazF-mt9 complex, benefiting from the strong charged attractions between the highly positively charged toxin (Figure 5C) and highly negatively charged antitoxin peptide. In the case of MazF-mt1, the pI values for the α3, α4, and α3–α4 helices are 3.4, 3.7, and 3.5, respectively, so the charged interactions do not significantly affect the affinity (Figure 5A). However, the binding of α4 would be energetically unfavorable due to the unusually strong cross-subunit loop interactions. Therefore, we could only cocrystallize the α3-peptide with MazF-mt1, but not the α3–α4 helices. Lastly, MazF-mt3 represents an intermediate case between the two above-mentioned members, with medium-strength loop interactions. The pI value of the α3–α4 fragment of MazE-mt3 (4.0) is close to that of α3 (3.8) but much lower than that of α4 (5.2) (Table 3). Here, the charged interactions and loop interference counterbalance each other, and the end result is a trade-off: the α3–α4 helices bind more strongly to the toxin and also interfere with the activity of the toxin (Figure 5B). Accordingly, this peptide cocrystallized with the toxin protein.

## 3. Discussion

Among the *M. tb* MazEF members, MazEF-mt2 and -mt8 are likely pseudogenes, as they were never successfully expressed in *E. coli* during our numerous tests. In the remaining MazEF members, mt-1/3/9 possessed long loops, and our laboratory solved the apo- or antitoxin-bound structures of all three. Through sequence and structure comparison, we classified these members into three types based on the β1–β2 loops: interlocked long loops with strong cross-subunit interactions (MazF-mt1), long loops with weak or medium cross-subunit interactions (MazF-mt9/mt3), and short loops without cross-subunit interactions (MazF-mt4-8). Due to different structural patterns, these members behaved distinctly. The binding affinity of MazE-mt9 was mainly conferred by the helix MazE-mt9/α4 because the antitoxin without this helix exhibited poor binding. In other words, other parts of the mt9 antitoxin barely contributed to the binding event. In the MazE-mt1 case, the presence of the interlocked loops of MazEF-mt1 required a large amount of energy to break apart (more than the binding energy of α4 alone could provide), preventing the direct binding of the last helix α4 [36]. Here, MazE-mt3 represents an intermediate example between MazEF-mt1 and mt9. The long loops MazF-mt3 only showed weak cross-subunit interactions, but they could not be easily disrupted by the binding of the α4-helix. However, contributions from the second-to-last helix (α3) compensated for the energy penalty to a certain extent, as shown by the fact that the two combined helices would inhibit the WT enzyme. Consequently, the peptide consisting of the last two helices showed a strong inhibitory effect comparable to that of the full-length MazE-mt3 against the truncated enzyme. Therefore, in terms of RNase cleavage activity, the WT MazF-mt3 enzyme behaved similarly to MazF-mt1, whereas the loop-truncated mutant behaved similarly to MazF-mt9. Likewise, MazF-mt6 behaved similarly to the truncated MazF-mt3 or full-length MazF-mt9 enzyme: the single peptide which corresponds to the last helix of its cognate MazE would lead to inhibitory effects comparable to that of the full-length antitoxin.

Taken together, MazEF-mt1/mt3/mt9/ systems utilize different strategies to regulate the toxin–antitoxin association despite their high sequence homologies, and this results from the interaction strengths of their long loops. Due to the sequence variation of several critical residues at the dimer interface, MazF-mt3 acquires the loops of medium strength that allow “conditional” binding, which binds the two C-terminal helices of MazE-mt3. In contrast, the intrinsically disordered loops of MazF-mt9 allow it to form a flexible/disordered region at the dimer interface and bind α4 of the antitoxin with a higher affinity. These different strategies are adopted by various MazEF TA systems to achieve efficient regulation over the cognate and heterologous interactions across the family, reflecting their functional needs accordingly. We speculate that the requirement of the last two helices of MazE-mt3 allows the toxin to differentiate its cognate antitoxin from MazE-mt9, ensuring that MazF-mt3 exclusively responds to its cognate antitoxin.

Conformational changes upon the formation of a toxin–antitoxin complex are not uncommon. Three types of scenarios have been reported, and we have previously summarized these scenarios according to their conformations before and after the binding of their cognate antitoxins [36]. MazF-mt3 represents scenario 3, where the cross-subunit loops remain open upon the binding of MazE-mt3. In scenario 2, the originally disordered loops of MazF-mt7 become ordered once MazE-mt7 becomes bound (PDBs 5XE2 and 5XE3) [38]. We currently have limited information as to how the binding of RNA substrates affects the functions of the toxins due to a lack of structural information of the RNA complexes. Elucidating these complex structures would greatly aid in our understanding of the structure–activity relationships and evolution of the *M. tb* MazEF families.

## 4. Materials and Methods

### 4.1. Materials

*Mycobacterium tuberculosis* H37Rv was donated by the Guangzhou Chest Hospital Laboratory Department (Guangzhou, China). Primers were synthesized by Ruiboxingke Biotechnology Co., Ltd. in Beijing, China. The 5′-FAM labeled substrate was synthesized by Huzhou Hippo Biotechnology Co., Ltd. (Huzhou, China). Peptides were synthesized by Qiangyao Biotechnology Co., Ltd. in Shanghai, China. Crystallization reagents were from Sigma-Aldrich (St. Louis, MO, USA), and other reagents were from ThermoFisher (Waltham, MA, USA).

### 4.2. Methods

#### 4.2.1. Cloning, Expression, and Purification of Proteins

*M. tb mazf-mt3* (Rv1991c), *maze-mt3* (Rv1991A), and *mazf-mt6* (Rv1102c) were amplified using genomic DNA of *M. tb* H37Rv as a template. Two forms of MazF-mt3 were used in this study, one of which was in the MBP-fused form for the crystallization purpose (the tag was removed prior to the crystallization), and the other for the activity assays with the N-terminal 6×His affinity tag. The former construct was cloned into a modified pCold-MBP vector (TaKaRa, DaLian, China) with an upstream 10×His tag and a downstream PreScission protease (Cytiva, Wilmington, DE, USA) cleavage site of MBP. The *mazf-mt3* PCR product and pCold-MBP were both digested with Sac| and Xho|, respectively. The ligation product was transformed into DH5α cells and verified by DNA sequencing. On the other hand, the 6×His versioned *mazf-mt3* gene was sub-cloned into the pET-28a vector (Merck, Darmstadt, Germany) after digestion by the Nde| and Xho| enzymes, followed by the subsequent ligation and transformation. The activity assays of MazF-mt6 involved the usage of TF-MazF-mt6 (i.e., the trigger-factor fused form) and *mazf-mt6* was sub-cloned into the pCold-TF vector (TaKaRa) by utilizing the Sac| and Hind||| restriction sites. The *mazf-mt3* mutants for the activity assays were generated by the QuikChange method (Stratagene, Bastrop, TX, USA) using the WT/pET-28a vector as the template. All clones were transformed into BL21 (DE3) cells and cultured at 37 °C in a 2 L LB medium supplemented with 50 μg/mL ampicillin or 30 μg/mL Kanamycin. Induction was performed overnight at 18 °C with a final concentration of 0.3 mM isopropyl β-D-1-thiogalactopyranoside (IPTG) when the OD_600_ reached 0.6–0.8.

MazF-mt3 was initially purified using Ni-NTA affinity chromatography column, followed by the overnight cleavage of the MBP tag with the PreScission protease with a mass ratio of 100:1 (Cytiva) in a cleavage buffer of 20 mM Tris-HCl pH 8.0, 150 mM NaCl, and 5% glycerol. The protein was dialyzed against 20 mM Tris-HCl pH 9.0 and 500 mM NaCl, and isolated using a MBP affinity chromatography column (Cytiva), by collecting of unbound fractions. The MBP binding buffer was 20 mM Tris-HCl pH 9.0 and 500 mM NaCl, and the elution buffer was 20 mM Tris-HCl pH 8.0, 100 mM NaCl, and 10 mM maltose. The His-tagged MazF-mt3, MazE-mt3, and TF-MazF-mt6 were purified using Ni-NTA affinity chromatography only and then dialyzed against 20 mM Tris-HCl pH 8.0, 150 mM NaCl, and 10% glycerol. The mutants were purified through the same protocol.

#### 4.2.2. Crystallization

The tag-removed MazF-mt3 protein was dialyzed against 20 mM Tris-HCl pH 9.0 and 500 mM NaCl and concentrated to 3 mg/mL. The MazE-mt3 (Leu46-Trp82) peptide was redissolved to 10 mg/mL in CAPSO pH 9.4, and then diluted to 2 mg/mL using a buffer of 20 mM Tris-HCl pH 9.0 and 500 mM NaCl. To obtain cocrystals, the two proteins MazF-mt3 and MazE-mt3 (Leu46-Trp82) were mixed at a 2:1 (molar ratio) with a final concentration of the toxin at 1 mg/mL, and incubated on ice for 30 min.

Prior to crystallization, the sample was centrifuged at 23,500× *g* for 10 min. Crystallization screening of the sample was conducted at room temperature using commercial screening kits from Hampton Research. After 2–3 days, diamond-shaped microcrystals of apo-MazF-mt3 appeared on the sitting drop plate using NaCl as the precipitant. Following the optimization, crystals were grown in a reservoir solution containing 1.5 M NaCl and 0.1 M sodium acetate pH 4.5. Crystals were harvested after approximately 1 week, supplemented with 20% glycerol (*v*/*v*) in the reservoir solution as a cryoprotectant, and flash-frozen in liquid nitrogen. Additionally, the MazEF-mt3 complex formed needle-shaped microcrystals on the sitting drop plate using ammonium sulfate as the precipitant. After optimization, final crystals were grown in a reservoir solution containing 1.2 M ammonium sulfate and 0.1 M MES pH 5.5. Crystals were harvested after approximately 3 weeks, supplemented with 20% glycerol (*v*/*v*) in the reservoir solution as a cryoprotectant and flash-frozen in liquid nitrogen.

#### 4.2.3. Data Collection and Structure Determination

Data collection from apo-MazF-mt3 and the complex was carried out at beamline 19U (BL19U) at the Shanghai Synchrotron Radiation Facility (SSRF, Shanghai, China), which was subsequently processed with the program HKL3000 [47,48].

The structure of the apo form was solved by molecular replacement using the Phaser program with the coordinates of the alphafold 2-predicted structure as the search model. On the basis of the solution, the model was further built manually with COOT according to the electron density map [49]. Multiple cycles of refinement alternating with model rebuilding were carried out with PHENIX.refine [50]. The final model was validated by MolProbity [51]. The structural figures were produced with PyMOL (www.pymol.org). All data collection and refinement statistics are presented in Table 1.

#### 4.2.4. In Vitro Cleavage Assays

To characterize the in vitro activity of MazF-mt3, we first explored optimal reaction conditions. After optimization, a typical 10-μL reaction system contained 0.5 μM MazF-mt3 and 1 μM RNA substrate in a reaction buffer of 20 mM Tris-HCl pH 8.0 and 150 mM NaCl. The reaction was conducted at 37 °C for 30 min, terminated by adding a 2× loading buffer, followed by heating at 95 °C for 5 min. Electrophoresis was performed on a 16.5% urea-PAGE gel containing 7 M urea at 110 V for 2 h. Imaging was conducted with a gel imaging system (ChemiDoc XRS+, Bio-Rad, Hercules, CA, USA), and quantification was measured using ImageJ 1.50i (NIH, Bethesda, MD, USA). The reaction efficiency was calculated as: % cleaved = product/(product + remaining substrate) × 100%. Error bars were calculated as standard deviation (s.d.) (n = 3 biological replicates).

For the inhibition experiment by the antitoxin, MazE-mt3 and MazF-mt3 were incubated at room temperature for 20 min to allow them to form a complex, which was then added to the above-mentioned reaction system.

#### 4.2.5. Thermal Shift Assay (TSA)

The proteins were in the buffer of 20 mM Tris-HCl pH 8.0 and 150 mM NaCl. The final concentrations of SYBR orange fluorescence dye (Invitrogen, Carlsbad, CA, USA) and MazF-mt3 (WT or mutants) were 2× and 8.2 μM, respectively. Three replicates were performed per group. After the reaction system was mixed in the 96-well PCR plate, the plate was put into the real-time fluorescence quantitative PCR instrument (BioRad). The program was set as follows: in the first step, a melting curve from 25 °C to 99 °C, with an increment of 0.5 °C every 5 s; and in the second step, 99.9 °C for 10 s. The fluorescence signals at 470/570 nm wavelengths for excitation and emission, respectively. Data analysis was conducted using OriginPro2024b (OriginLab, Northampton County, PA, USA).

## Figures and Tables

**Table 1:** Data collection and refinement statistics.

	MazF-mt3-Apo (9IKD)	MazF-mt3-MazE (8ZWS)
Data Collection		
Space group	*P*4_3_2_1_2	*P*3_1_21
Cell dimensions		
a, b, c (Å)	110.8, 110.8, 271.2	84.7, 84.7, 101.8
α, β, γ (°)	90, 90, 90	90, 90, 120
Resolution (Å)	75.27–3.32 (3.50–3.32) *	73.39–3.27 (3.45–3.27) *
*R* *_merge_*	0.16 (1.31)	0.16 (0.99)
*I/*σ*_I_*	18.4 (2.8)	14.8 (2.8)
Completeness (%)	92.7 (78.8)	94.2 (81.0)
Redundancy	24.2 (21.3)	14.1 (12.4)
Refinement		
Resolution (Å)	75.27–3.32 (3.47–3.32)*	73.39–3.27 (4.12–3.27) *
No. reflections	23,795	6445
*R*_work_/*R*_free_ (%)	24.2/27.9	23.9/26.1
No. atoms		
Protein	9020	2690
*B*-factors (Å^2^)		
Protein	109.8	83.3
R.m.s. deviations		
Bond lengths (Å)	0.003	0.002
Bond angles (°)	0.61	0.48
Ramachandran		
Favored (%)	97.51	96.80
Outliers (%)	0.33	0

* Values in parentheses are for highest-resolution shell.

**Table 2:** The toxin–antitoxin hydrogen-bonding interactions observed in the TA complex.

Antitoxin (Chain I)	Toxin (Chain A)	Toxin (Chain B)
Thr47 (OG1)		Pro34′ (O)
Asp51 (OD1)		Arg39′ (N)
Glu55 (N)		Arg39′ (NH2)
Asp61 (OD1)	Asn51 (ND2)	
Asp61 (OD2)	Thr49 (OG1)	
Glu62 (OE2)	Arg25 (NH2)	
Glu62 (OE2)	Arg25 (NE)	
Glu64 (OE1)	Asn77 (ND2)	
Glu64 (OE2)	Asn77 (ND2)	
Glu64 (OE2)	Thr79 (OG1)	
Asn70 (OD1)	Thr83 (N)	
Asn70 (ND2)	Thr83 (O)	

**Table 3:** The isoelectric points of the *M. tb* MazEF systems.

TA Systems	MazE				Full-Length MazF
	α3	α4	α3–α4	Full-Length	
mt1	3.43	3.70	3.47	4.37	9.10
mt3	5.20	3.83	4.00	4.63	8.40
mt9	6.80	3.70	4.44	5.10	10.40

## Data Availability

All data relevant to this study are supplied in the manuscript are available from the corresponding author upon request. Coordinates and structure factors are deposited in the Protein Data Bank with the PDB entries: 9IKD and 8ZWS.

## Supplementary Materials

The supporting information can be downloaded at: https://www.mdpi.com/article/10.3390/ijms25179630/s1.
