# Supplementary material for: Long Dynamic β1–β2 Loops in M. tb MazF Toxins Affect the Interaction Modes and Strengths of the Toxin–Antitoxin Pairs
Source: Int J Mol Sci. 2024 Sep 5;25(17):9630. doi: 10.3390/ijms25179630 (PMC11394972; doi:10.3390/ijms25179630)
Supplement: Supplementary file 1 [file ijms-25-09630-s001.zip › ijms-3174239-supplementary.pdf]

Supplementary Information

ARTICLE

Long Dynamic β1-β2 Loops in *M. tb* MazF Toxins Affect the Interaction Modes and Strengths of the Toxin-Antitoxin Pairs

Ziyun Tang[1,2,†], Pengcheng Jiang[1,2,†], and Wei Xie[1,2,*]

[1]MOE Key Laboratory of Gene Function and Regulation, State Key Laboratory for Biocontrol, School of Life Sciences, The Sun Yat-Sen University, Guangzhou, Guangdong, People's Republic of China.

[2]Innovation center for evolutionary synthetic biology, school of life sciences, The Sun Yat-Sen University, Guangzhou, Guangdong, People's Republic of China.

[†]These authors contributed equally to this work.

* Corresponding author

E-mail: xiewei6@mail.sysu.edu.cn (WX)

Table of Contents

Figure S1. Structural comparison of the two subunits of the MazF-mt3 dimer. 3

Figure S2. The close-up view of the dimer interface at the C-termini of MazF-mt3 (corresponding to Figure 1D). 4

Figure S3. The RNA-cleavage activity tests of the WT MazF-mt3 enzyme. 5

Figure S4. The expression and purification profiles of MazF-mt3 and mutants. 6

Figure S5. The TSA assay results of the WT MazF-mt3 and mutants for stability evaluation. 7

Figure S6. The crystal packing patterns of the half complex. 8

Figure S7. Structural superimposition of the apo-MazF-mt3 dimer (PDB 9IKD) with the MazEF-mt3 ternary complex (PDB 8ZWS).crystal packing patterns of the half complex. 9

Figure S8. The cleavage activities of MazF-mt3 in the presence of MazE-mt3. 10

Figure S9. The relative activities of MazF-mt3 (△16-22) mutant in the presence of inhibiting peptides. 11

Figure S10. The cleavage activities of MazF-mt6 in the presence of the inhibiting peptide MazE-mt6/α4. 12

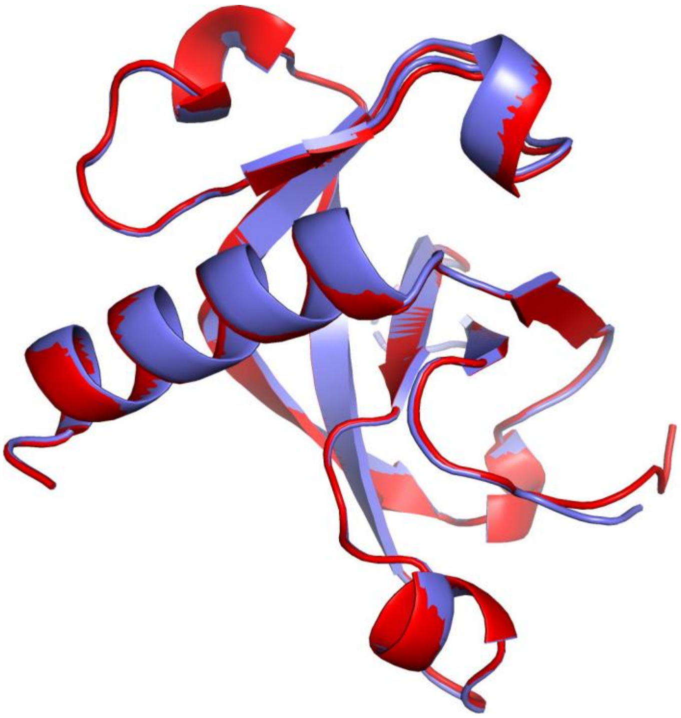

Figure S1. Structural comparison of the two subunits of the MazF-mt3 dimer. The two subunits were colored red and slate, respectively, and were shown in the ribbon rendition.

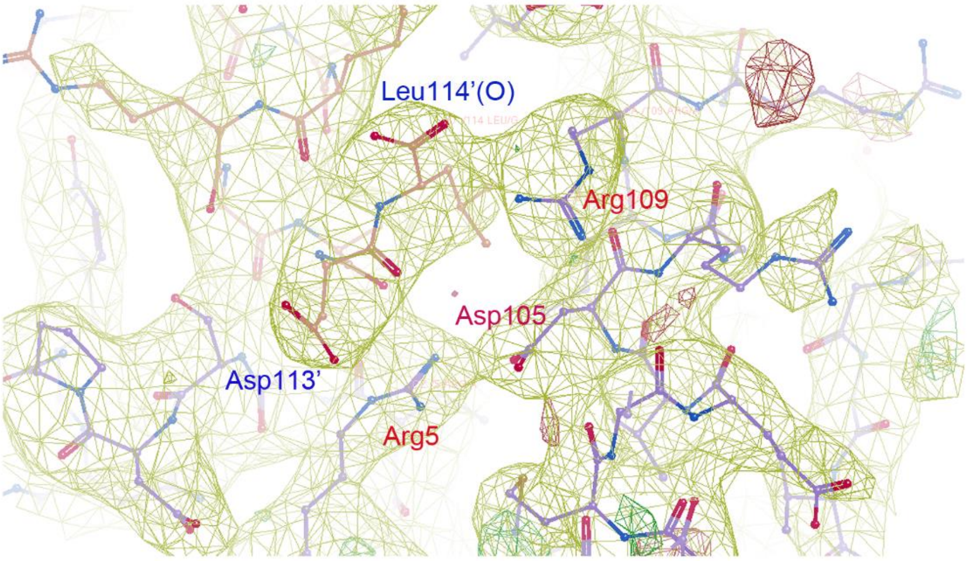

Figure S2. The close-up view of the dimer interface at the C-termini of MazF-mt3 (corresponding to Figure 1D). The 2Fo-Fc electron density was contoured at 1 σ by coot.

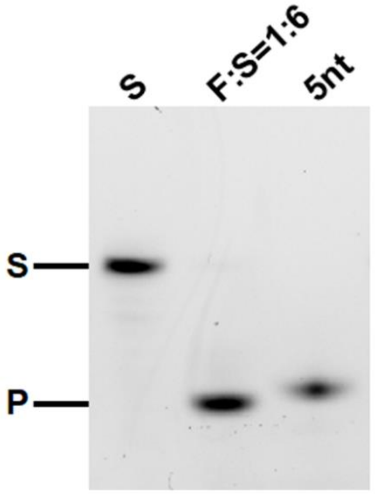

Figure S3. The RNA-cleavage activity tests of the WT MazF-mt3 enzyme. S: substrate; P: product. The middle lane represented the reaction with an enzyme/ substrate molar ratio of 1:6, while the right lane was a 5-nt RNA marker.

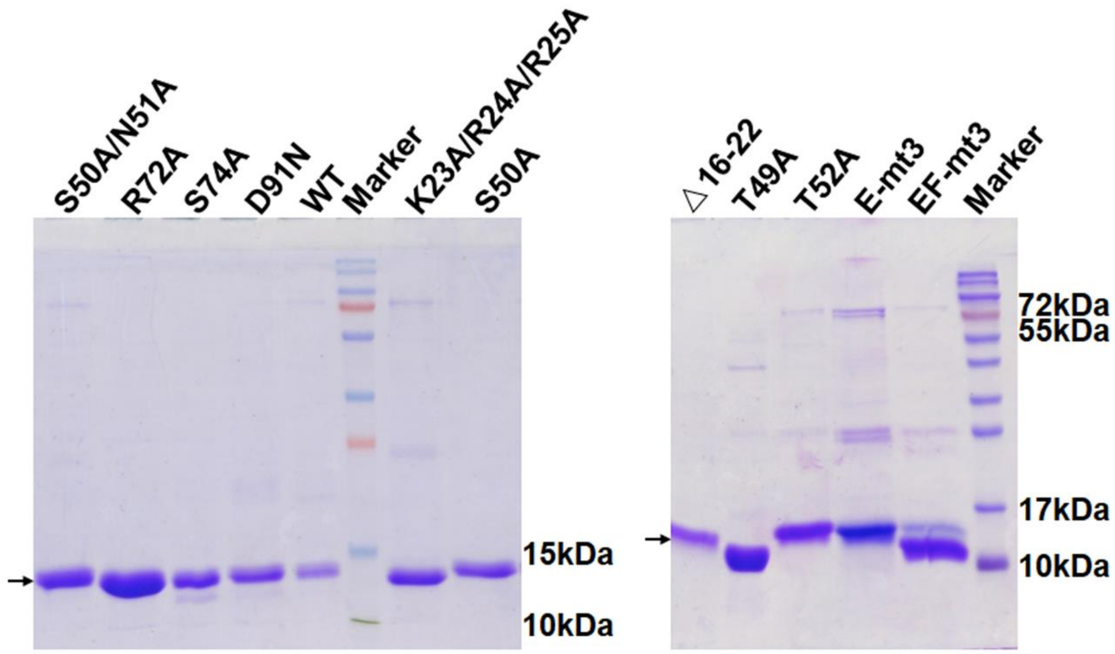

Figure S4. The expression and purification profiles of MazF-mt3 and mutants. The arrow marked the position of the target protein.

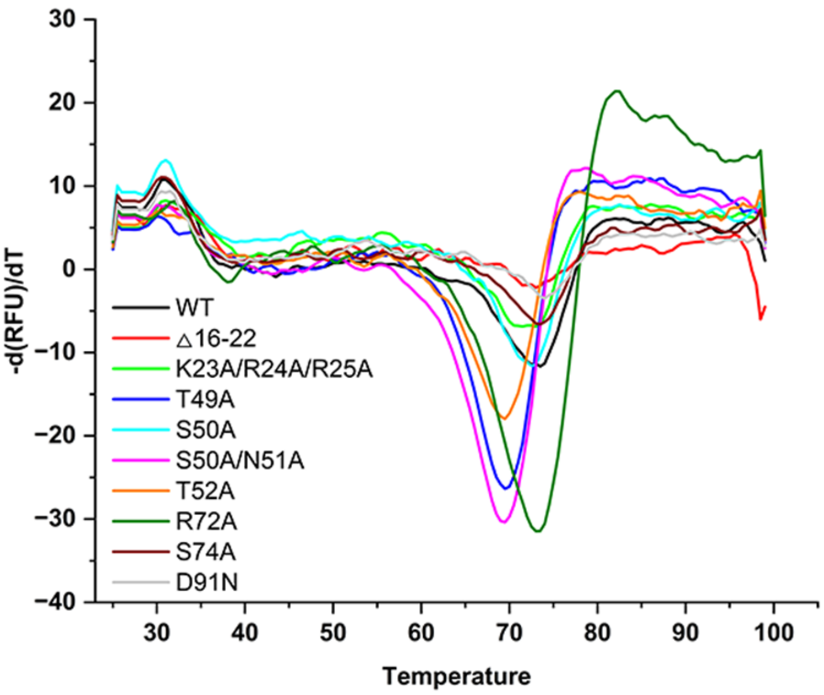

Figure S5. The TSA assay results of the WT MazF-mt3 and mutants for stability evaluation. Triple replicates were carried out. The curve is the average of the triple replicates. The horizontal axis indicated temperature while the vertical axis indicated relative fluorescence units (R.F.U.).

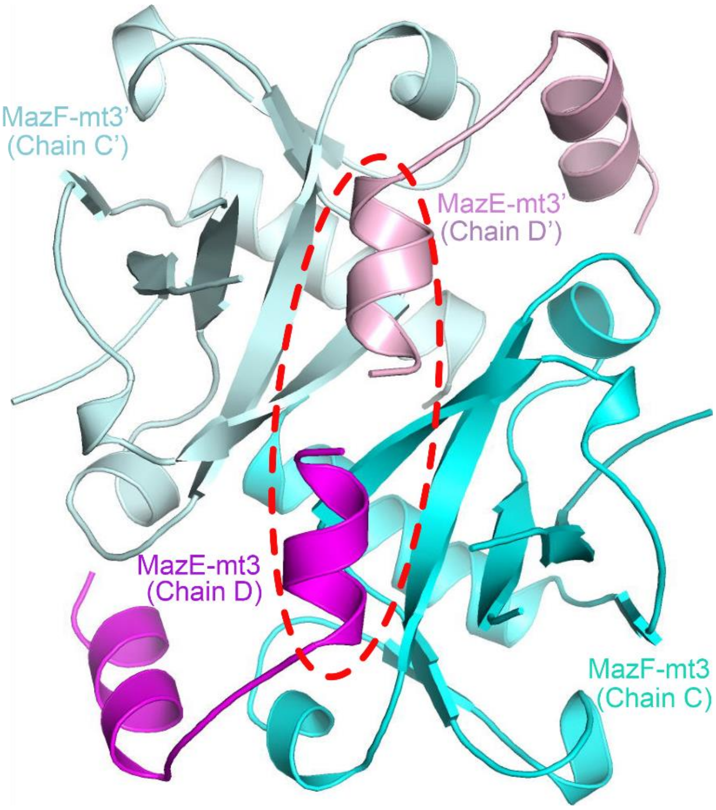

Figure S6. The crystal packing patterns of the half complex. The MazF (chain C) and MazE peptide (chain D) resolved in the "half complex" were shown in cyan and magenta, respectively, while their symmetry-related counterparts were colored pale cyan (chain C') and light pink (chain D'), respectively. The two MazE peptides circled by the red oval coincide with the two-fold crystallographic symmetry axis, and make "end-to-end" crystal contacts.

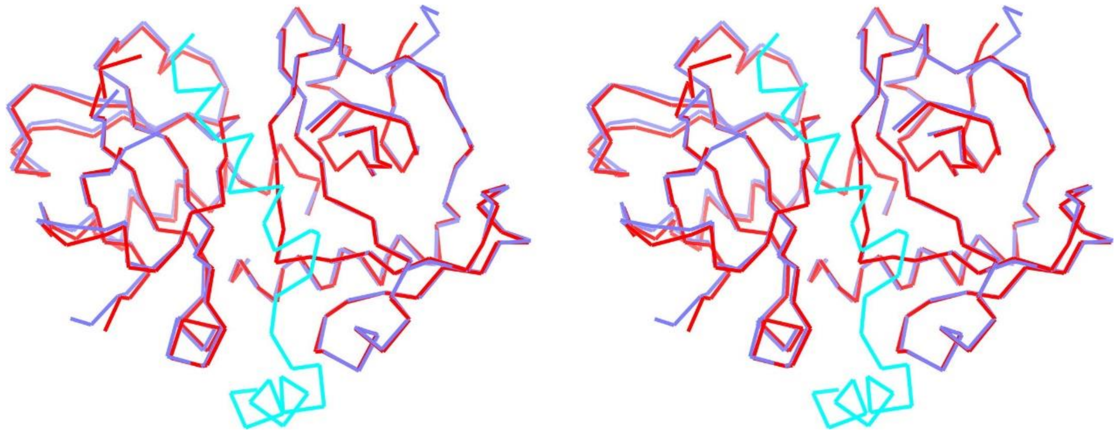

Figure S7. Structural superimposition of the apo-MazF-mt3 dimer (PDB 9IKD) with the MazEF-mt3 ternary complex (PDB 8ZWS). The two MazF dimer structures were colored red and slate, respectively, while the MazE peptide was colored cyan. All structures were rendered in the backbone traces.

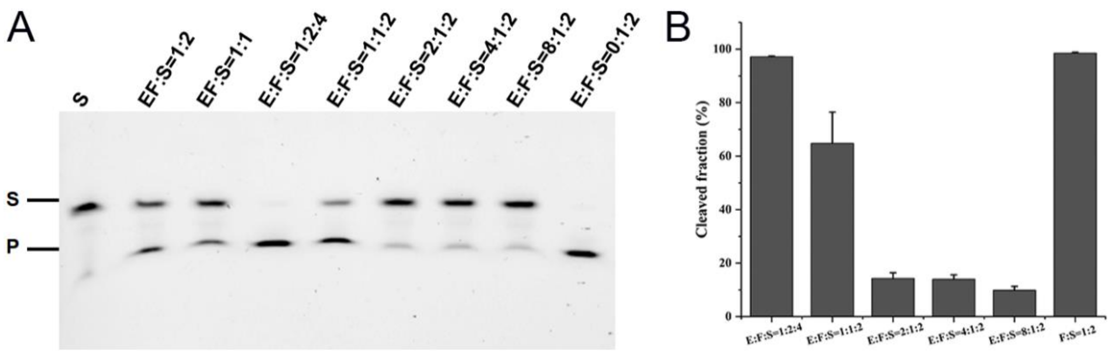

Figure S8. The cleavage activities of MazF-mt3 in the presence of MazE-mt3. The numbers above the lanes indicated the molar ratios of peptide/toxin/RNA. (A) The gel-electrophoresis of the cleavage assays. “EF” in lanes 2 and 3 represented the co-expressed MazEF-mt3 complex while the rest lanes employed MazF-mt3 mixed with increasing concentrations of the antitoxin (separate expression for each). (B) The quantification of the enzymatic activities, with the vertical axis indicating the cleaved fractions of the substrate.

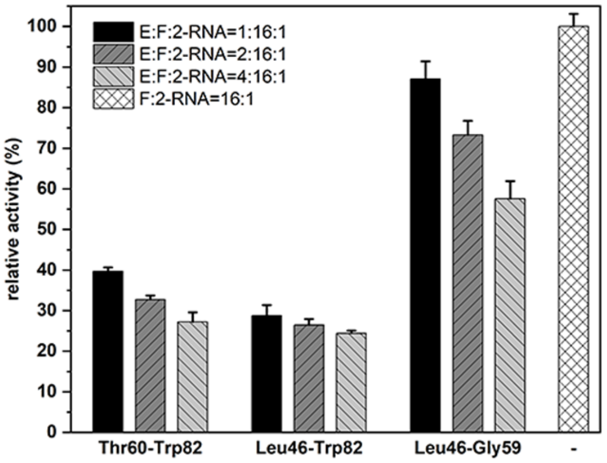

Figure S9. The relative activities of MazF-mt3 (△16-22) mutant in the presence of inhibiting peptides. The quantification of the enzymatic activities, with the vertical axis indicating the relatively cleaved fractions of the substrate. Error bars are standard deviation (s.d.) (n = 3 biological replicates).

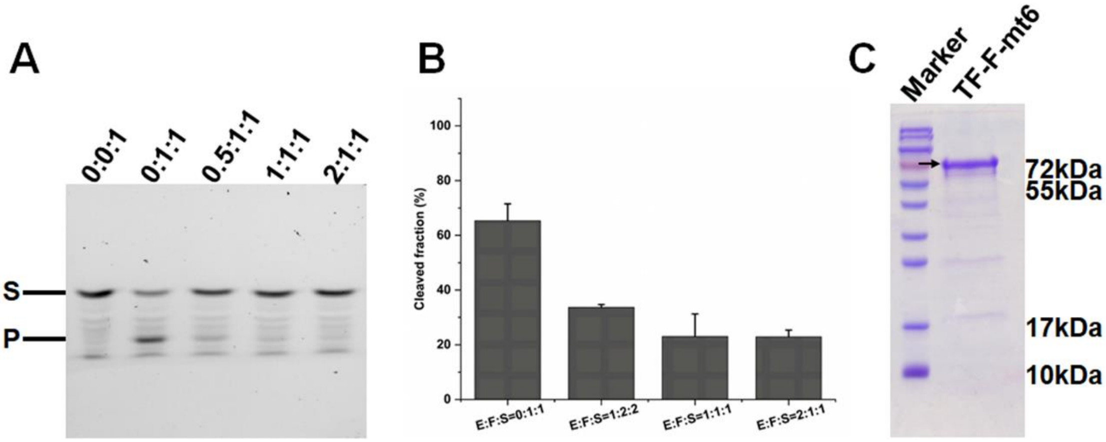

Figure S10. The cleavage activities of MazF-mt6 in the presence of the inhibiting peptide MazE-mt6/α4. The numbers above the lanes indicated the molar ratios of peptide/toxin/RNA. (A) The gel-electrophoresis of the cleavage assays. (B) The quantification of the enzymatic activities, with the vertical axis indicating the cleaved fractions of the substrate. (C) The expression and purification profile of MazF-mt6.
